# Bibliographical Notices

**Published:** 1851-02

**Authors:** 


### BIBLIOGRAPHICAL NOTICES.

*Transactions of the American Medical Association, instituted* 1847. Vol. III. Philadelphia, 1850. pp. 499.

Concluded.

REPORT ON MEDICAL LITERATURE.

This Report, from the pen of Dr. Alfred Stillé, Chairman of the Committee, discusses with marked ability the Medical Journalism of the United States, exposing with candor and directness its defects, while it commends what is valuable and important in the original contributions of American physicians during the past year. The committee claim, we think with justice, no small degree of merit for our medical periodical literature, and consider American journals, at the present day, as "indispensable to writers upon the practice of medicine and surgery." An appendix to the report contains a list of all original articles of interest published during the year, forming a valuable table for reference, and one which we hope will be continued in future reports.

The remarks of the committee upon the "critical department of the medical journals" are marked by discrimination and good sense, and would be read with profit by that class of writers who figure in this department. The following high tribute is paid to the reviewers, and we shall not call in question its truthfulness:

"The 'Reviews,' as distinguished from the 'Bibliographical Notices," generally present a clear and faithful account of the works to which they relate, with occasional comments; as the opinions of the author and reviewer coincide or diverge. It very rarely happens, in such elaborate analyses, that the pen of criticism appears to be guided by improper feelings, whether of impartiality or dislike. They seem, in the great majority of instanees, to be dictated by a spirit of justice, and that they are so is rendered probable by the striking unanimity of sentiment expressed in various journals respecting the same work. Exceptions to this statement may undoubtedly be discovered, yet we regard it as a fact, no less certain than praiseworthy, that the medical critics of the United States are remarkably independent in thought and expression. They display but little of that tendency so evident in British periodicals, to proportion the eulogy of a book to the reputation of its author, and to frown upon a work, however meritorious, to which an unknown name is attached. In this matter American critics unquestionably feel the influence of liberal institutions, and appear to be all but unconscious of either hopes or fears inspired by the authors whom they judge."

A catalogue of the books published in the United States during the year, accompanies the report, with short notices of each of the *original* works.

This summary of the progress of medical literature during the year, furnishes the committee with a text for some general reflections and suggestions upon the importance of a national literature to the medical character of the United States.

The causes of the imperfections of our native literature are pointed out with a clearness and a felicity of style rarely excelled. Imperfect education, deficient mental training, a vitiated language, and a "debased popular idiom," are mentioned as prominent obstacles to the attainment of that high excellence to which American medical writers should aspire. The necessity for institutions of learning of a higher grade than any of those which now exist, is thus enforced:

"The true exponents of the literature of an epoch or profession are not the most popular works, but those from which popular works are extracted, those which are fullest of invention and discovery, those to which the historian will afterwards point as the monuments of their time. No argument need be adduced to prove that these can only be created by a system of exceptional culture above, and different from what is suited to the average wants of the profession; that there must be seats of professional learning whither all may resort who have exhausted the ordinary means of instruction and yet thirst for higher knowledge. At present, no such institutions exist in this country; and hence nearly all writers who have adorned the native literature of our science have been compelled to seek abroad the culture which they vainly wished for at home, and, as an inevitable result, to adopt opinions, and habits of expression which have tended to make their works pale copies of transatlantic originals, when they should have been stamped with the characteristics of the American mind. An elevated, distinctive and permanent medical literature cannot, we think, be hoped for until some one of our schools shall adopt a much higher system of instruction than has yet been thought of, and, instead of looking to minor institutions for the measure of its advancement, look upwards only to the sublime purpose of education, receive into its bosom those alone who are fitted by their accomplishments and their ambition to honor the profession, and resolve that the American student shall be perfected on American soil.

On such a purpose, steadily maintained, and supported by every expedient that is calculated to develop and mature the talent of those who enter upon medical pursuits, must the hopes of our professional literature after all repose, and not upon any narrow-minded attachment to American literature for its own sake merely. It is not to be pampered because it is a native growth, but to be fostered because it is good or capable of becoming so. It would ill become us to imitate the people of China and Japan, who pride themselves upon their stunted and effeminate civilization, and look with contempt upon the sciences and arts of the outside barbarians. A feeling of nationality in literature is, within just limits, a mighty stimulus to the production of works which extend the empire of the mind, but, carried beyond a certain point, becomes a high treason to the universal republic of letters, and 'confines to a sect what was meant for mankind.' While, then, every lover of his country must be ambitious of raising to its glory the only imperishable memorial that human skill can construct, a national literature, he ought to remember that the work will crumble of its own weakness, unless its foundations are deeply and securely laid in that knowledge which is of no one time or place, but is the common product of all ages, and of the whole civilized world."

The subject of an international copy-right law in its connection with the present state of American literature, is discussed with ability and perspicuity. Upon the advantage to be derived from such a law the committee remark:

"The benefits of such a law would be incalculable, springing from the one glorious consequence, that through it the thinking and therefore the governing minds of two great nations, common in their origin and language, would be brought directly into contact and fused together, strengthening one another, by communion, rivalry, and example, and would with this redoubled strength reach and maintain with ease the vantage-ground of civilization against all the world besides. Commerce may knit together nations, but only in their material interests, binding them with the bonds of selfishness; but a free intellectual commerce is the noblest of all bonds, for it knits the souls of men.

A law such as would secure the rights which we assume to be absolute and inherent, is perhaps the surest expedient that could be devised for promoting our native literature. Not only would it enable publishers to offer a fair remuneration to our authors, but it would also have the effect of causing a number of foreign works to appear first in this country; and since the sphere of influence presented by the millions of American readers is so far superior to any other, many a foreigner of genius and even of established reputation would speedily be numbered amongst our adopted citizens. Grafted thus with the strongest offshoots of the parent tree, American literature would rapidly attain a degree of luxuriance and fruitfulness, which is quite impossible under the present system of its culture, or rather neglect. But the benefits would be reciprocal; our writers would become English writers, and find an attentive and appreciating audience among a people who even now receive them gladly, and award to those who deserve it no scanty meed of praise."

Our space forbids more extended comments upon this able paper, but we cannot leave it without expressing the high gratification which it has afforded us. Whether viewed as a specimen of elegant composition, or as the vehicle of just and practical views. upon the subject treated of, we would commend it to the consideration of the profession.

### REPORT ON PUBLIC HYGIENE.

This report is confined mainly to that department of hygiene having relation to epidemics and diseases, especially "the sources of typhus fever, and the means suited to their extinction." The Chairman, Dr. Joseph M. Smith, of New York, regarding the origin of typhus to be from human excretions, points out, with his usual ability, the kinds and quantities of these matters concerned in its production. A critical analyisis of the proximate principles present in the pulmonary and cutaneous excretions as revealed by recent experiments, is presented, and to the animal matter in these is ascribed, the idio-miasma or typhus poison. The quantity of this eliminated from the entire amount of matters discharged from the lungs, taken in a given time has been proximately arrived at, by the investigations of Ancell of London. The report thus sums up the results:

"Adopting the chemical views of Liebig, and pursuing his method of research, this gentlemen has ingeniously attempted 'to exhibit the various modes in which the equivalents of the component parts of the *ingesta* are eliminated from the body, and to ascertain the elementary constitution of the solid, fluid, and gaseous compounds into which they are distributed in the *egesta*.' The subject of Mr. Ancell's experiment was a healthy man, who, in twenty-four hours, took as *ingesta* 96 oz., and received, by estimate, into the system by the lungs, stomach, and skin, 32 oz. 23 dwt. 4 grs. of oxygen, making a total of matter received into the body of 129 oz. 3 dwt. 4 grs. The weight of the individual was the same at the end as at the beginning of the experiment. The egesta during the time were found to be fæces 5 oz. 10 dwt.; urine, 42 oz. and matters eliminated from the lungs and skin, consisting of water, carbonic acid, sweat, tears, animal matter, &c., 81 oz. 13 dwt. 4 grs. in all, making a quantity equal in weight to the matters introduced into the system.

The great difference in the amount of the excretions by the lungs and skin, in Mr. Ancell's estimate, and the quantities stated by former experimentalists arises from his bringing into his calculation the amount of oxygen taken into the system through the lungs, stomach and skin, and expelled from the pulmonary and cutaneous surfaces in combination with carbon and hydrogen, forming carbonic acid and water. Subtract the oxygen in question, and the quantity of matters excreted by the lungs and skin is reduced to 48 oz. 14 dwt., a sum differing but little from the estimates given, as before noticed, by Hartman aud Von Gorter.

On carefully analyzing the table in which the results of Mr. Ancell's inquiries are exhibited, it is found that of the 81 oz. 13 dwt. 4 grs. of cutaneous and pulmonary excretions, 42 oz. 13 dwt. 10 grs. consistof water, 9 dwt. of dry materials are found in the secretions of the skin, and consist of *animal matter* 8 dwt., and salts 1 dwt., which added to the *animal matter* exhaled with the pulmonary vapor, give, exclusive of the salts, 11 dwt., 7 grs.: that is, the amount of *animal matter* emitted with the excrementitious discharges through the lungs and skin in twenty-four hours. This amount probably exceeds the usual quantity, and consequently, to avoid exaggeration, we shall assume 10 dwt. as the normal daily average."

The circumstances under which the effete matters of the human body may be productive of typhus, both in private dwellings and public institutions, is a subject of exceeding interest; and is treated by the committee in an instructive manner.

The influence of over-crowded, illy ventilated rooms,.occupied by filthy tenants, in the production of typhus, is also forcibly depicted, and the foul agencies thus developed are considered amply sufficient to produce the disease, without resort to the doctrine of specific contagion, although the influence of this cause is not denied in particular cases.

Many other interesting views upon the etiology of typhus are set forth, which our limits will not allow us to notice. The report, so far as it develops the one topic selected by the chairman, is a masterly production, and we have only to regret that the same mind did not take a wider range of inquiry into this neglected department of our science.

Appended to the report is a paper on the "Sanitary condition of Massachusetts," by Dr. Edward Jarvis, a member of the committee. This paper presents us with a history of the public acts of Massachusetts in reference to the sanitary question, showing that State to be in advance of any other in the Confederacy, in the care which she has extended over the physical condition of her people. It is to the untiring energy and perseverance of a few men, amongst whom the name of the writer of this report stands conspicuous, that the enviable position of this State upon the sanitary question, is mainly attributable. This fact should afford encouragement to those interested in other States, to persevere in this labor of science and benevolence.

Dr. Jarvis has instituted a comparison between the mortality of the States of New York and Massachusetts, and between the cities of New York and Boston, as revealed by the registration reports of the two States, which will be found exceedingly interesting to those engaged in such enquiries.

The comparative mortality of the cities and suburban districts of the states, is also discussed, so far as the published reports furnish the data, though it is admitted that as yet these are not sufficiently full to arrive at satisfactory results on this point.

It is inferred, however, from the facts which have been collected, and from the corroborating testimony of the more accurate and complete returns of mortality in England and Wales, that "life is fostered less successfully in the city than in the country."

The full returns of the mortality in England and Wales, as published in the fifth report of the Registrar General, accompany Dr. Jarvis' paper, and upon them he has made some observations and comparisons which will be found valuable to the statistical student. The report closes with the following sentiment in regard to the importance of hygiene as a branch of medical science.

The few facts that are known are, to this great subject, like the few pieces of ore or coal that the proprietor finds in an unexplored field. They indicate that much more is to be discovered, and the importance of farther examination. A sanitary survey of this and of every other State would, I fear, demonstrate an inequality in the distribution of life in various places and among various classes of people, such as few either of the philanthropists or political economists now suspect. And, when these facts shall be demonstrated, the way will be open to the discovery of many of the causes of disease and death, some of which certainly, many of which probably, can be removed, and the health of man thereby be increased and his life prolonged.

With this view we fully accord, and trust that whatever changes the American Medical Association may make in the arrangements of its committees, or in the features of its organization, it will still retain the subject of public hygiene, as one deserving of an annual examination by a competent committee.

"Report on the Hygienic characteristics of New Orleans, by J. C. Simonds, M. D.," is the title of another paper appended to the report on Public Hygiene.

Dr. Simonds has presented many facts of interest in regard to the hygienic condition of New Orleans, tending to develope sources of the ill health which prevails there, and which it would be wise in his fellow-citizens seriously to ponder.

The drainage of the city is represented as exceedingly defective, although measures are in progress which it is believed will remedy this evil in the course of a few years.

The supply of water is shamefully restricted, being "*monopolized by a close corporation* dispensing it very sparingly and at most exorbitant rates, which have this year been increased over twenty-eight per cent. to families." The poor man, we are told, pays the same price for this necessary of life, as the wealthy citizen, though he consumes less. Dr. Simonds knows of but ONE *free hydrant*, and that in a location that renders it of but little use! Well may Dr. S. exclaim—

"How much longer our citizens will submit to this misused monopoly cannot be determined; but that they have a right, paramount to any restrictions that may be imposed by a legislature, to procure for themselves an abundant supply of this necessary of life, scarcely admits of question."

In making statistical tables of deaths, in New Orleans, Dr. S. has experienced the common difficulty of defective returns, based upon the reports of sextons, and false or ambiguous certificates from physicians of all classes, both real and pretended. He has, however, presented the profession with some novel and interesting views on the principles of classification of diseases, which are well worthy the attention of statisticians.

The mortuatory tables which accompany the report, evince on the part of the writer great care and industry, although we fear the labor bestowed on their preparation, will yield him no other return than the consciousness of having performed a public duty, and the thanks of that small band of his professional brethren who are devoted to similar pursuits. The crowd bestow no praise on such "dry details."

*Report on Adulterated Drugs, Medicines, and Chemicals, &c.* Dr. R. M. Huston, *Chairman.*

The important subject entrusted to this Committee, has been faithfully investigated, and a mass of interesting facts, which would probably not otherwise have been revealed, has been the consequence. The inquiry is divided into two sections—the first relating to the adulterations of foreign, and the second to that of domestic drugs.

The late Act of Congress, in regard to the former, is said by the Committee to have been productive of much benefit—both by the actual prevention of dishonest traffic, and by stamping a practice which was once carried on with impunity, with the seal of public condemnation.

In regard to "home adulteration," which it has been feared by some, was on the increase, since the late passage of the law above referred to, the Committee, after special investigation, inform us, that in their opinion "there does not appear to be any *increase*, but probably a *diminution* in the domestic sophistication of drugs."

The grounds upon which this assertion is made, will be found interesting to enquirers upon the subject, and the Committee deserve much credit for their industry in collecting the facts.

Many articles still subject to adulteration, are reported upon, and the profession are thus placed on their guard in relation to them.

The following are the conclusions arrived at by the Committee on this branch of the subject:

"1st. That the wholesale druggists in the large cities, equally in the South and West as in the Eastern States, who are not specially engaged in selling nostrums, either as proprietors or agents, conduct their business on fair and honorable principles. As a general rule, they buy their choice chemicals from those who manufacture them, and either import other articles, or get them directly from those who do, and are always disposed to supply good articles to customers who are willing to pay a remunerating price. At the same time, many of this class keep inferior articles, which they dispose of for a corresponding price to physicians and storekeepers who insist on buying at reduced rates.

2d. That the inferior and adulterated drugs are chiefly disposed of in the southern and western portions of the United States—to the physicians and people residing in the small towns and villages, and sparsely populated districts. That in the large cities, particularly in the Atlantic States, bad drugs are, as a very general rule, dispensed only by inferior apothecaries."

The report closes with some important recommendations in regard to the sophistication and sale of spurious drugs at home, which are specially addressed to medical societies.

### *Report on Indigenous Medical Botany.*

This report from the pen of Dr. Eli Ives, of New Haven, is confined to the author's personal experience of the virtues of a few individual plants, not before enumerated by the preceding Committees.

The medical profession are perhaps more indebted to Dr. Ives, than any other living physician, for his researches into the medicinal virtues of native plants, and hence this report, restricted as it is, will be found to possess much practical interest. A simple enumeration of the articles mentioned would carry us beyond our already extended limits, and we can, therefore, only commend the paper of Dr. Ives to the general perusal of physicians.

*Report of the Committee on Surgery.*

This paper occupies sixty pages of the Transactions, and though confined to American Surgery, takes a more extended retrospect than a single year would furnish. It is from the pen of the venerable President of the Association.

The subject of Anæsthesia occupies a prominent place in the report, and a full account is given of the experience of American surgeons within the year, both as it relates to the value of anæsthetic agents in general, and to the relative value of particular articles. Without reviewing the facts here, we can merely state that the result of the enquiries of the Committee fully confirms previous experience, as to the great importance of this new class of agents in the practice of Surgery, although a difference of opinion still exists, as to which of the articles employed preference should be given.

The subject of Cancerous Tumors, referred by a previous Committee to the consideration of this, is fully discussed, and a mass of valuable facts are spread out before the profession, touching the question of the curability of cancer, whether by operative procedures, or by remedial measures.

The results, so far as operations are concerned, are, we think, sufficiently discouraging, to deter surgeons from recommending extirpation of cancerous tumors of the breast, and other really malignant growths, as a means of cure. We would gladly transfer to our pages the experience of some of the most eminent surgeons of the country upon this point; but we must be content with recommending those interested in the inquiry to consult the volume for themselves. The subjects of Aneurism, Injuries of the Head, Gun-shot Wounds, Fractures, Hernia, Lithotomy, Ovariotomy, Excision of the Uterus, Cataract, with a variety of other facts collected by the Committee, form altogether a valuable compend of the present state of American Surgery. From a review of this able paper, we fear that the rage for operating which prevails to no small extent, over the surgical world, to the injury of the caüse of true science, is still too rife amongst American surgeons; and if we should venture a criticism on the report before us, it would be that this spirit is not sufficiently discouraged by the Committee.

With these remarks we must bring our notice of the Transactions to a close. Their strictly national character, and the strong desire that we feel to present a fair analysis of them to our readers, will, we trust, prove a sufficient apology for the length we have given to their notice. That they are, as a whole, highly creditable to the Association, and that they manifest an evident progress in Medical Science in our country, will, we think, be admitted by every candid reader. The reports of the various Committees are thorough and practical, and the spirit with which the proceedings recorded in the volume appear to have been conducted, augurs well for the future prosperity and usefulness of the Association.

1. *Introductory to the Course of Lectures on the Theory and Practice of Medicine, in the University of Pennsylvania.* By GEORGE B. WOOD, M. D. Delivered October 11th, 1850.
2. *Impediments to the Study of Medicine; a Lecture, introductory to the Course of Practice of Medicine.* By J. K. MITCHELL, M. D., Professor in the Jefferson Medical College. Delivered the 18th of November, 1850.
3. *Lecture Introductory to the Course of Materia Medica and Pharmacy, in the University of Pennsylvania.* By JOSEPH CARSON, M. D., Professor of Materia Medica and Pharmacy. Delivered October 10, 1850.
4. *An Introductory Lecture, delivered at the opening of the thirty-first Session of the Medical College of Ohio, November 4th,* 1850. By JOHN BELL, M. D., Professor of the Theory and Practice of Medicine.
5. *An Introductory Lecture, preliminary to a Course on the Institutes of Medicine, delivered on the 9th of October,* 1850, *before the Medical Class of the University of Pennsylvania.* By SAMUEL JACKSON, M. D., Professor of the Institutes of Medicine.
6. *An Introductory Address to the Class of the Medical Department of Pennsylvania College, Session* 1850—51. By WASHINGTON L. ATLEE, M. D., Professor of Medical Chemistry.
7. *Introductory Address delivered before the Class of the Medical Department of the Saint Louis University, Session of* 1850—51. By M. M. PALLEN, M. D., Professor of Obstetrics and the Diseases of Women and Children.

We are indebted to Professors Wood, Mitchell, Carson, Jackson, Bell, Atlee and Pallen, for copies of their Introductory Lectures delivered at the opening of the current sessions in their respective schools. All of these addresses are well-written and instructive contributions to our medical literature; several of them have a special present interest, from recent changes in the professional position of the lecturers.

That Dr. WOOD'S Introductory to his first Course on the Theory and Practice of Medicine in the University of Pennsylvania, has been received with lively interest by all who are connected with American medicine, we need hardly say. The importance of the chair, the distinguished reputations which have already illustrated it, and the high anticipations formed from Dr. Wood's career in another station, could hardly fail to make him on such an occasion, the 'observed of observers.' We are sure our readers will be gratified by having placed before them the personal allusions with which the new professor assumed the honored mantle, to which all American physicians of every interest and association, must look with something of attachment and of veneration.

"You are aware that the professorship I now fill became vacant soon after the close of the last session, by the resignation of Dr. Chapman, whose failing strength induced him to withdraw from its onerous duties, after a career of honor and usefulness, never surpassed in the medical history of this country. In the movements which afterwards took place in reference to the filling of the vacancy, I was quite passive. An honorable ambition might suggest that the Professorship of the Practice, occupied as it had been by men so illustrious, held a somewhat more elevated place in public estimation than that of Materia Medica; and the consideration was not without weight, that in the former a new field was opened, calculated to stimulate energies and industry, which, without some fresh excitement, might perhaps slumber in the hebetude of increasing age. On the other hand, however, were the reflections, that with my existing duties I was quite familiar; that to fulfil them it was only necessary to keep up with the advancing tide of knowledge; that rest rather than increased labor was suited to my time of life, or at least soon would be; and that, in making a change, I might be abandoning a position to which sufficient trial had shown me to be in some degree adequate, for another in which I might not prove equally useful, or give equal satisfaction. The motives for a change were insufficient to outweigh those of a contrary character; and having, therefore, no personal ends to answer, I had no other wish, in reference to the future arrangements, than that they should be such as would conduce to the advantages of the school.

To my colleagues and to all others concerned, when interrogated on the subject, I freely made known these sentiments; declaring truly that I was personally indifferent whether, in the approaching election, another or myself should be chosen, that I was desirous only that the true interests of the school should be consulted, and that, not being the best judge of my own qualifications, I should take no step in the matter, but leave it altogether to the wisdom of those in whose charge the Institution was placed. Should they believe that I could best serve the school in the department of Materia Medica, I would cheerfully persevere in my former path of labor, and cordially greet any new colleague whom the Trustees might elect to the vacant professorship. Should it, on the contrary, be thought that I could do better service in the practical chair, I would acquiesce in the decision, and exert myself to the extent of my capacity, to fulfil its various and burthensome requisitions.

Under these circumstances, the choice, as you are aware, fell on myself; and I should not be doing justice to my feelings on the occasion, if I did not confess to you that this mark of confidence was in the highest degree gratifying to me, though involving much of labor, of sacrifice, and of misgiving on my part.

And now, gentlemen, you will, I hope, be disposed to judge me leniently in my new sphere of duty; and, should the result of my efforts fall short of your expectations, or of the just demands of the position, you will at least acquit me of undue presumption.

There is another consideration, which, in justice to myself, I must beg of you to bear in mind, in forming your estimate of the ensuing course of lectures. You will necessarily compare them with the past. Even those of you who have not enjoyed the opportunity of listening to my predecessors, have yet heard, and still hear the prolonged echo of their praises, and have in your own mind formed a standard of excellence, in connexion with this chair, by which you will naturally be disposed to judge its present occupant. But remember, gentlemen, that these were men the most eminent in their profession whom this country has produced, whose names are set like priceless gems in our history, and who, standing in the morning light of medical teaching on this continent, loom in magnificent hues on your admiring vision. Drs. Morgan, Kuhn, Rush, Barton, and Chapman, have successively held the professorship of the Theory and Practice of Medicine in this school. Here, in this very spot, is enthroned the reputation of these distinguished men, and receives willing homage from the young thronging aspirations that surround it. A successor approaches the place illuminated by such great recollections, and is for the moment invested, in the imagination of the spectator, with the lingering effulgence. You will admit with me, gentlemen, that this is a most trying position. In the cold presence of reality, the unsubstantial glory soon fades away, and the new-comer stands in the severe outlines of truth, with no coloring from the rainbow hues of fancy; nay, dimmed and beclouded to eyes that had been dazzled by the previous brightness. This trial I am now to undergo. May I not ask of you, gentlemen, to close your eyes firmly on the past; and, in judging of my efforts, if indisposed to a partial indulgence, at least to view them in their own true light, and not through the medium of an impairing contrast."

The subject proper of Dr. Wood's address, is an introduction to his course—the Theory and Practice of Medicine. Its importance and its difficulties are depicted; a general outline of the lecturer's plan for the conduct of his course is sketched, with an allusion to his resources for making it as 'demonstrative as possible;' and the lecture is concluded with an eloquent and feeling tribute to the merits of his immediate predecessor.

Dr. J. K. Mitchell's address, now before us, is an admirable exposition of 'the impediments to the study of Medicine,'—which we should desire to see placed in the hands of every person about to enter upon it. Dr. Mitchell takes the highest ground on the subject of the preliminary qualifications which should be exacted from the student of medicine; and, while expressing a generous sympathy with the enterprise and perseverance, which, in spite of early deficiencies, will sometimes deservedly attain the honors of our profession, he offers a forcible and earnest protest against "encouraging persons to enter upon the study of medicine without suitable preliminary learning." He well observes that

"In the earlier periods of our national existence, this evil was a necessary consequence of the state of society. Refined and well-educated men could not often consent to bury themselves in the woods to earn amidst hardships and privations a scanty subsistence. The higher qualities of the profession could not be often used, and were more seldom properly appreciated. Indeed, few students of medicine could find means of obtaining a good preliminary education, or of following out in foreign universities a complete system of instruction. Hence society was compelled to take the best accessible adviser, and habit reconciled it to what was a necessary evil. *It is so no longer! &c. &c.*"

This well-timed appeal in favor of a high standard of pre-requisites for the study of medicine, is the opening chapter to an excellent volume of general advice to the student about to engage in the pursuit of professional knowledge. Altogether, Dr. Mitchell's Introductory will add not a little to his reputation as a sound and eloquent teacher.

The maiden effort of Dr. Carson, in the chair to which he has lately been elevated with a unanimity of approbation not often elicited, is in every way equal to the anticipations of his friends and worthy of his new position. A learned and judicious sketch of the history of his department—the Materia Medica—is appropriately followed by an impressive and eloquent delineation of its just claims to importance among the branches of medicine. We give a brief extract from the conclusion of the lecture, as a specimen of the very pleasing and elegant style of the new professor.

"Besides the efficiency communicated by a thorough knowledge of the Materia Medica, in all its details and bearings, there are other points of in view which it must be regarded. It is inseparable from the possession of general information.

The history of drugs is in a measure associated with the history of nations; as objects of care and culture they are identified with the races of the country in which they are produced: thus pepper and cubebs are associated with the habits and mode of life of the Malays, rhubarb in like manner with the Tartars, senna with the Arabs, opium with the Turks, and camphor with the Chinese. Among civilized nations the trade in them has occasioned struggles and encounters which constitute a portion of the revolutionary annals of the world. The discovery and supremacy of the Portuguese in India, their trade in costly gums, spices, and valuable products, the cession to the Dutch and their monopoly, and the downfall of Holland's power before the giant arm of England, or the exclusive traffic of the Spaniards in the products of the South American Colonies and subsequent failure to maintain it, from internal political resistance and foreign aggression, will serve as an illustration of the association referred to. From histories of discoveries and conquest, from books of travels, are to be gleaned valuable facts pertinent to the subject."

Of another maiden effort, with which we have been favored, we may say, that it has been anticipated with the same friendly interest, and welcomed with the same satisfaction, as the last. Dr. Bell bore with him to the new and distant field of labor to which he has been called, the warm wishes of a large circle of friends and of the profession generally, in Philadelphia, and, while we greatly regret his loss here, we look forward with pleasure to the prospect of his success and increased usefulness elsewhere. The address now before us was delivered on the fourth of November, last, in Cincinnati, on the occasion of Dr. Bell's assuming the chair of Theory and Practice of Medicine, in the Medical College of Ohio. It is a spirited and vigorous production, graced with a fancy and animation of style, which we were not entirely prepared for—and, we need hardly add, distinguished by an elevated tone on the subject of medical education. Such language as the following, is, indeed, of the right stamp:

"It should be the aim of the different schools of the country, in a spirit of generous rivalry, to raise the standard while increasing the means of medical education. They must carefully reject all the expedients to swell the numbers of their classes, which smack of the petty, and not always conscientious trafficker, rather than the elevation of true science, and the sensitiveness of refined literature. Encouragements held out to the illiterate, the unstable, and it may be the more unworthy still, to study medicine, are premiums given to ignorance and indolence. Many a good mechanic and thrifty farmer are lost in making them bad doctors. Genius requires no such hot-bed culture. It is a vigorous plant and will grow in a scanty soil and a bleak atmosphere, and bear fruit under an ungenial sun. It will show itself soon enough to insure all needful aid, without its being debased by ostentatious and selfish patronage and eleemosynary support."

Dr. JACKSON'S Introductory is characterized by the freshness of thought and language, and the elevated earnestness of strain, with which we have been so often attracted and instructed in his occasional addresses. The topics of his present discourse are —"the evidences that prove conclusively the existence of order and laws in Physiology and Medicine, as well established as in the positive sciences." An eloquent and just picture is presented of the pretensions of medicine to the title of an exact science—a picture which recalled to us another of the same subject by the same hand, not less eloquent, but more discouraging, upon which we had occasion to comment several years ago.* Lectures like the present are, we think, of the best tendency. They inspire the student with the enthusiasm for his profession, which is the only basis of success and usefulness; and, notwithstanding a redundancy of technicality, which is perhaps out of place on such an occasion, Dr. Jackson's Introductory strikes us as being in the happiest style of this kind of effort.

From the Pennsylvania College, we have to acknowledge the receipt of the Introductory of Dr. W. L. ATLEE, Professor of Medical Chemistry—a well-conceived and well-written address, devoted to the unpretending, but appropriate subjects connected with the scholastic course of medical students. We have seen no better manual of advice suitable to the occasion.

Dr. PALLEN'S Introductory, delivered before the Medical Class of the Saint Louis University, is a very striking and thoughtful production on the true value of observation in Medicine, from which we should desire to extract largely, but for the length which this article has reached. We cordially recommend it as an address well worthy of close perusal.

In closing our notices of the Introductories of the present season, we take the occasion to express our regret at not having been favored with a larger number of these addresses, from other schools than those of Philadelphia. It is our desire to maintain

* Dr. Jackson's Introductory, delivered November, 1841.

the Examiner in every respect free from local bias or subserviency to sectional interests; and with this view, we may here remark, that we should be happy to present *clinical reports* from schools at a distance that may be disposed to send them to us. Published in a city and state eminently central in situation, and *national* in feeling, the Examiner will, we trust, bear this character in its relations to American medicine.

---

*The Medical Student's Guide in Extracting Teeth:* with numerous cases in the Surgical branch of Dentistry; with illustrations. By S. S. HORNOR, Practical Dentist. Philadelphia. Lindsay & Blakiston, 1851.

This little manual, which the author tells us is intended for the medical student more especially, contains much practical information that is valuable. It will be found particularly useful to country practitioners, upon whom the duty of extracting teeth very often devolves. It contains a description of the indispensable instruments, and their mode of application, together with numerous cases illustrative of the various operations. We recommend it cordially to those for whose use it was prepared.

